# Experimental Investigation into the Preparation Process of Graphene-Reinforced Aluminum Matrix Composites by Friction Stirring Processing

**DOI:** 10.3390/ma17163918

**Published:** 2024-08-07

**Authors:** Gaohong Chen, Mei Yu, Hongrui Dong, Jianhua Liu

**Affiliations:** 1 Beijing Institute of Aeronautical Materials, Beijing 100095, China; 2 School of Materials Science and Engineering, Beihang University, Beijing 100191, China; 3 College of Mechanical and Electrical Engineering, Nanjing University of Aeronautics and Astronautics, Nanjing 210016, China

**Keywords:** graphene, aluminum, friction stirring, interface reaction, dispersion

## Abstract

Graphene has been considered an ideal reinforcement in aluminum alloys with its high Young’s modulus and fracture strength, which greatly expands the application range of aluminum alloys. However, the dispersion of graphene and the interfacial reaction between graphene and the aluminum matrix limit its application due to elevated temperature. Friction stirring processing (FSP) is regarded as a promising technique to prepare metal matrix composites at lower temperatures. In this paper, FSP was used to prepare graphene-nanoplates-reinforced aluminum composites (GNPs/Al). The corresponding effects of the process parameters and graphene content on GNPs/Al were thoroughly studied. The results showed that plastic strain, heat input, and graphene content were the key influencing factors. Large degrees of plastic strain can enhance the dispersion of graphene by increasing the number of stirring passes and the ratio of stirring to welding velocity, thereby improving the strength of GNPs/Al. Low heat input restricts the plastic flow of graphene in the matrix, whereas excessive heat input can promote interfacial reactions and lead to the formation of a more brittle phase, Al_4_C_3_. This is primarily associated with the stirring velocity and welding velocity. High graphene content levels can improve the material strength by refining the grain size, improving the load transfer ability, and acting as a precipitate to prevent dislocation movement. These findings make a contribution to the development of advanced aluminum alloys with graphene reinforcement, offering broader application potential in industries.

## 1. Introduction

In recent years, metal matrix composites (MMCs) have attracted much attention in the aerospace industry due to their unique physical and mechanical properties [1,2]. Metal matrix composites are formulated on the foundation of metals and their alloys and are further fortified through external or internal growth techniques. Among different nanoscale reinforcements, graphene has promising potential to change the mechanical, electrical, thermal, and tribological properties of metal alloys [3,4]. For example, a graphene-reinforced aluminum matrix composite is a metal matrix composite with superior performance, obtained by using aluminum and its alloys as a matrix and adding graphene as a reinforcement. The addition of a small amount of graphene can significantly improve the tensile strength and micro-hardness of the aluminum matrix [5,6,7]. However, challenges persist in the preparation of GNPs/Al, encompassing issues such as the dispersion of graphene within the aluminum matrix, the wettability between graphene and the aluminum matrix, and the interfacial reaction between graphene and the aluminum matrix [3].

Some research work has been conducted to solve the above problems. The main preparation processes include powder metallurgy, melt metallurgy, chemical synthesis, and electrodeposition [8,9,10]. Zhang et al. [11] successfully prepared GNPs/5083Al composites using powder metallurgy technology, and corresponding process steps include ball milling, hot pressing sintering, and hot extrusion. It was found that the yield strength of GNPs/5083Al increased by 50% by adding 1.0% of graphene, and good graphene dispersion was obtained. However, there was a brittle phase, Al_4_C_3_, in the matrix, indicating that the interface reaction occurred between graphene and the aluminum matrix. Based on the melt metallurgy, An et al. [12] prepared graphene-reinforced aluminum composites by adding a mixture of GNFs, TiH_2_, and aluminum powder into molten aluminum and stirring thoroughly. The result showed that its mechanical properties were greatly improved. Liu et al. [13] prepared Ni-NPs@GNP/6061Al by chemical synthesis, which is an ultrafine Ni nanoparticles (NPs)-decorated graphene hybrid (indicated with Ni-NPs@GNP). It was revealed that the yield strength and tensile strength of 0.7 wt% Ni-NPs@GNP/6061Al composites were increased by 75% and 30%, respectively. In summary, all the above methods need to be conducted at elevated temperatures. As a result, the interface reaction between graphene and aluminum cannot be avoided, which often leads to the formation of the brittle phase Al_4_C_3_ and affects the properties of composites.

To suppress the interface reaction under elevated temperature, some research has shown that friction stirring, as a solid-state bonding technique, has the potential to obtain composites with uniformly dispersed reinforcing phases and excellent mechanical properties [14,15,16]. Zhang et al. [8] prepared a graphene–aluminum powder with a mass fraction of 1.0% using a combination process of powder metallurgy and a friction stirring process. It was found that graphene was uniformly dispersed in the aluminum matrix, and just a small amount of Al_4_C_3_ was found in the matrix. The yield strength of the composites increased by 30.5%. Chen et al. [17] prepared graphene-reinforced magnesium-based composites using a combination of melt metallurgy and friction stirring processes. The results showed that graphene exhibited agglomeration and poor dispersion during melt metallurgy, while good dispersion was obtained after a multi-pass friction stirring process. In summary, the friction stirring process is an effective method to obtain good dispersion with large degrees of plastic strain under solid-phase states. However, preparing metal matrix composites with a friction stirring process is still a new method. There is still a lack of enough data about the effect of process parameters on the preparation of GNPs/Al, and their corresponding mechanisms are also unclear.

In this paper, the effects of friction stirring process parameters (stirring pass and the ratio of stirring/welding velocity) and graphene content on the mechanical properties of GNPs/Al are studied. The stirring pass varies from one to four, the ratio of stirring/welding velocity varies from 1000/200 to 1000/40, and the graphene content varies from 0 to 2%. Then, the corresponding microstructure evolution is analyzed and discussed by OM and TEM observation from different aspects, including the evolution of mechanical properties, grain size, and interface.

## 2. Materials and Experiment

### 2.1. Materials

The substrate sheet used was a 1060 commercially pure aluminum plate under annealed temper conditions, whose chemical composition and mechanical properties are shown in Table 1 and Table 2, respectively.

**Figure 1:**
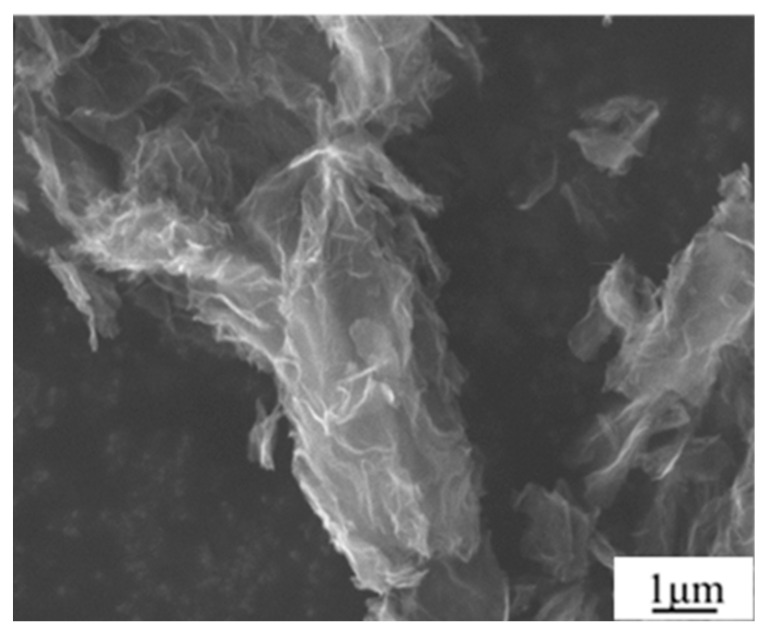
The microstructure character of graphene reinforcement.

The reinforcement material used was reduced graphene oxide with a thickness of 5–20 nm, produced by the Beijing Institute of Aeronautical Materials, whose microstructure is shown in Figure 1. Graphene oxide was prepared using the improved Hummers method, using flake graphite, potassium permanganate, concentrated sulfuric acid, and nitric acid as raw materials. The procedure adopted is indicated here as follows. First, mix all raw materials at room temperature; then, raise the temperature to 30–50 °C, allowing acid and potassium permanganate to intercalate into the flake graphite. Then, heat it to 95–98 °C to undergo an oxidation reaction, forming oxidized graphite (many layers), and then peel it off. Then, wash it multiple times with hydrogen peroxide, hydrochloric acid, and pure water to remove metal ions and acid from it. After that, spray dry to obtain graphene oxide powder. Graphene exhibits a lamellar structure with wrinkles on its surface. Moreover, graphene layers exhibit certain agglomeration phenomena. This may be due to the fact that a lamellar structure has a very large specific surface area (about 400~500 m^2^/g) and surface energy. From a thermodynamic perspective, graphene spontaneously clusters in the direction where surface energy decreases.

### 2.2. Preparation of GNPs/Al

**Figure 2:**
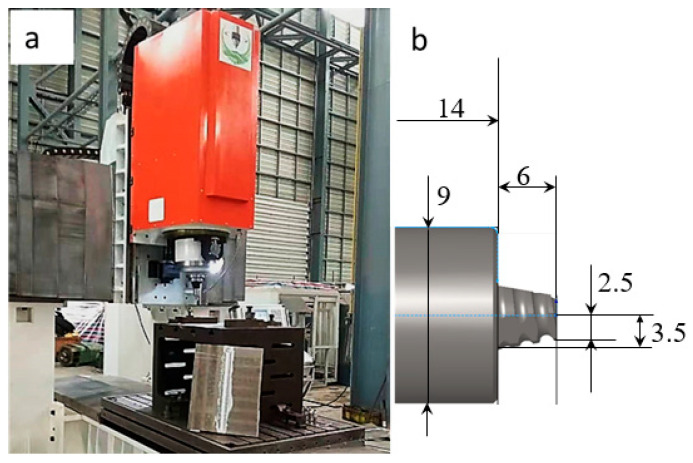
The FSM-TS1106 friction stirring welding machine. (**a**) Friction stirring welding machine; (**b**) a schematic of the stirring pin (unit: mm).

GNPs/Al were prepared with a friction stirring process. The equipment used was the FSM-TS1106 friction stirring welding machine, which is provided by Firstway Welding Technology Co., Ltd. in Shanghai, China, as shown in Figure 2a. The equipment mainly includes two parts: the lower part is the clamping platform, and the upper part is the friction stirring platform. The stirring head used is depicted in Figure 2b, whose shoulder diameter is Φ18 mm. The stirring pin is in the form of a conical thread, with an upper diameter of Φ5 mm, a lower diameter of Φ7 mm, a length of 6 mm, and a left-hand spiral configuration.

**Figure 3:**
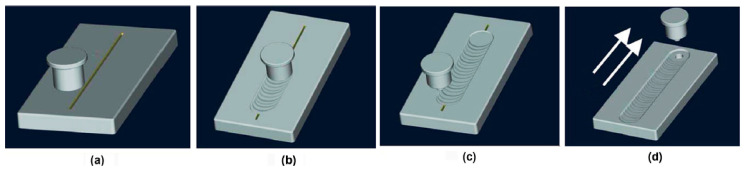
A schematic of the preparation process of GNPs/Al with FSP. (**a**) Original position, (**b**) welding process, (**c**) returning to the original position and (**d**) a repeated pass.

A schematic of the preparation process of the GNPs/Al is shown in Figure 3, and the main process parameters include stirring velocity, welding velocity, and stirring passes. Firstly, the stirring head rotates and embeds into the substrate plate, whose rotation velocity is called stirring velocity. Then, the stirring head moves from one end to the other end, and this movement velocity is called welding velocity, which is marked as one pass. After that, the stirring head is pulled out of the substrate, and it returns back to the original position. Finally, the above operation repeats to complete multiple passes.

In addition, graphene content is also an important influence factor on the mechanical properties of GNPs/Al. Therefore, based on the above process, the graphene content and the effects of FSP parameters on the preparation of the GNPs/Al were studied, as shown in Table 3. The pass numbers included 1, 2, 3, and 4; the stirring/welding velocity included 1000/200, 400/40, and 1000/40; and the graphene content included 0, 0.5, 1, and 2 vol.%. Among them, the ratio of the stirring/welding velocities were 5, 10, and 25.

### 2.3. Mechanical Property Tests

**Figure 4:**
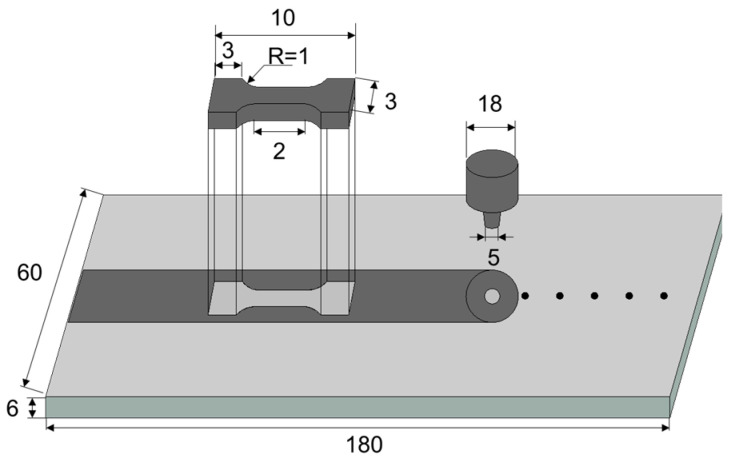
The cutting position and size of the specimen (unit: mm).

The mechanical properties of the GNPs/Al under different parameters were tested using uniaxial tensile tests. The corresponding cutting position and specimen size are shown in Figure 4. The specimens were cut along the welding direction in the stirring zone. Before testing, 1200 # sandpaper was used to polish the specimen surface to eliminate scratches and other defects, as well as avoid errors caused by stress concentration. After that, uniaxial tensile tests were conducted and repeated three times.

### 2.4. Microstructure Observation

Optical microscope (OM) and transmission electron microscope (TEM) tests were conducted to reveal the microstructure evolution under different conditions. The OM tests were conducted with a Carl Zeiss metallographic microscope at The Analysis & Testing Center, Beihang University, Beijing, China. The sample for the OM tests was firstly polished with 3000 # sandpaper—then, with a diamond polishing paste. After that, a 3% volume fraction HF solution was used for corrosion treatment, with a corrosion time of 2 min. After that, it could be used for the OM tests.

The TEM tests were conducted with JEM 2100F, produced by JEOL Ltd. (Tokyo, Japan), to analyze the characteristics of the phase. Firstly, the specimen was cut into a thin sheet with a thickness of 0.5 mm. Then, it was polished to 70 μm with sandpaper, and a circular disc with a diameter of 3 mm was punched out. Afterwards, the disc was thinned and perforated with a mixed solution of 70% methanol and 30% nitric acid as the electrolyte. The working temperature was −30 °C, and the working voltage was 20 V.

## 3. Results and Discussion

### 3.1. Mechanical Properties

**Figure 5:**
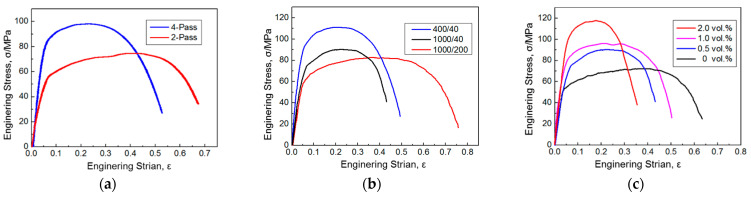
The engineering stress/engineering strain curves under different preparation process parameters. (**a**) Stirring passes, (**b**) the ratio of the stirring/welding velocity, and (**c**) the graphene content.

The mechanical properties of GNPs/Al under different preparation parameters are shown in Figure 5. As shown in Figure 5a, with the increase in stirring pass, the strength increases and the elongation decreases. The ultimate strength of GNPs/Al under four passes is 99.8 MPa, which is 42.5% higher than that under two passes, but the elongation decreases. With the increase in stirring passes, graphene is gradually and uniformly dispersed in the aluminum matrix, and the interface bonding strength between graphene and the aluminum matrix is also improved, further enhancing the strengthening effect of graphene. Therefore, the strength of GNPs/Al increases with the increase in stirring passes.

Figure 5b shows the effect of the ratio of stirring/welding velocity (RSWV) on the mechanical properties of GNPs/Al. The ultimate tensile strengths under 1000/200, 400/40, and 1000/40 are 81 MPa, 111 MPa, and 94 MPa, respectively. The ultimate strength first increases and then decreases with the increase in RSWV. When the RSWV is small, it reduces the plastic flow ability of an aluminum matrix during friction stirring, which makes it difficult to improve the dispersion of graphene. However, a large RSWV can cause excessive heat input, which can result in an excessive interfacial reaction between graphene and the aluminum matrix, produce more of the brittle phase Al_4_C_3_, and decrease the mechanical properties of GNPs/Al.

Figure 5c shows the effect of graphene content on the mechanical properties of GNPs/Al. With the increase in graphene content, the ultimate strength of the GNPs/Al increases, and the elongation decreases. When the graphene content is 2.0 vol.%, the ultimate strength of the sample reaches 119.2 MPa, which is 25.4% higher than the sample with 1.0 vol.% graphene content and 70.2% higher than the composites without graphene. The graphene not only refines the grain size but also improves the load transfer ability. Therefore, the strength of the sample can increase with the increase in graphene content. However, the elongation decreases with the increase in graphene content, which is mainly because the dispersion of graphene in composites decreases with the increase in graphene volume fraction. Agglomerated graphene acts as a crack source during the stretching process, causing a decrease in material elongation.

### 3.2. Microstructure Evolution

#### 3.2.1. Grain Evolution under Different Conditions

**Figure 6:**
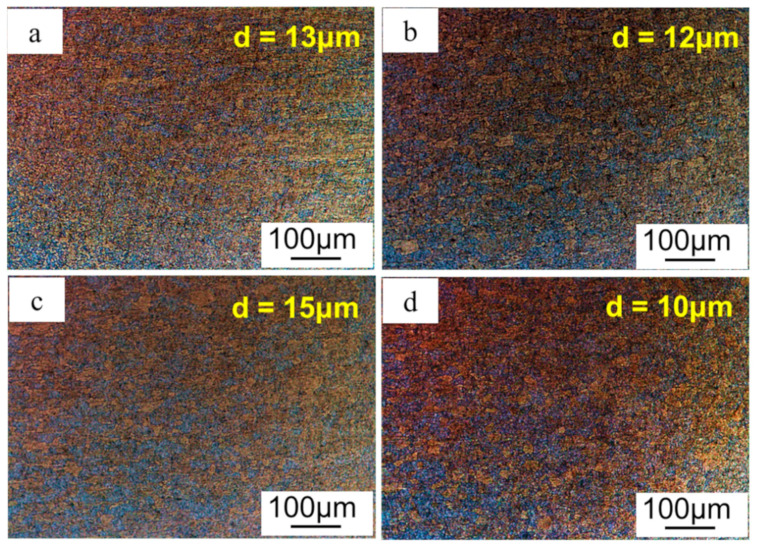
Microstructure of GNPs/Al under different passes: (**a**) one pass, (**b**) two passes, (**c**) three passes, and (**d**) four passes.

Grain size impacts grain boundary-related strength and influences the evolution of dislocation [18]. Firstly, the effect of stirring pass on the microstructure of GNPs/Al was characterized under the conditions of graphene content of 0.5 vol.% and RSWV of 1000/200, as shown in Figure 6. It was found that fine equiaxed grain could be obtained after friction stirring under the action of large degrees of plastic strain. The grain sizes under one, two, three, and four passes were 13 μm, 12 μm, 15 μm, and 10 μm, respectively. This shows that the grain size variation was not significant—within 10–15 μm. As mentioned earlier, the stirring zone underwent severe plastic deformation, and dynamic recrystallization occurred during the friction stirring process. Under different passes, the grain growth and the dynamic recrystallization due to severe plastic strain reached dynamic equilibrium. Therefore, the grain size of the stirring zone did not change with the variation in stirring passes.

**Figure 7:**
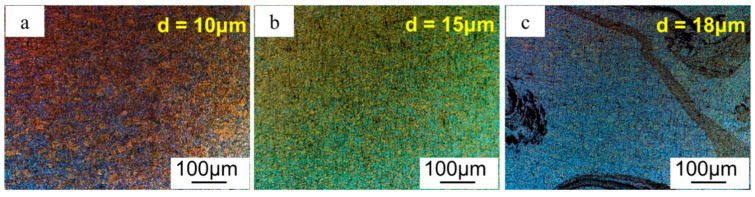
Microstructure of GNPs/Al under different ratios: (**a**) 1000/200, (**b**) 400/40, and (**c**) 1000/40.

The microstructure of GNPs/Al under different ratios of stirring/welding velocity is shown in Figure 7. Corresponding grain sizes under 1000/200, 400/40, and 1000/40 were 10 μm, 15 μm and 18 μm, respectively. The grain size at stirring zone increased with the increase in ratios of stirring/welding velocity. As mentioned earlier, the stirring zone underwent severe plastic deformation, and dynamic recrystallization occured during the friction stirring process. As the ratio of stirring/welding velocity increases, it can cause excessive heat input due to friction between the stirring head and the substrate, which can easily lead to grain coarsening in the stirring zone.

**Figure 8:**
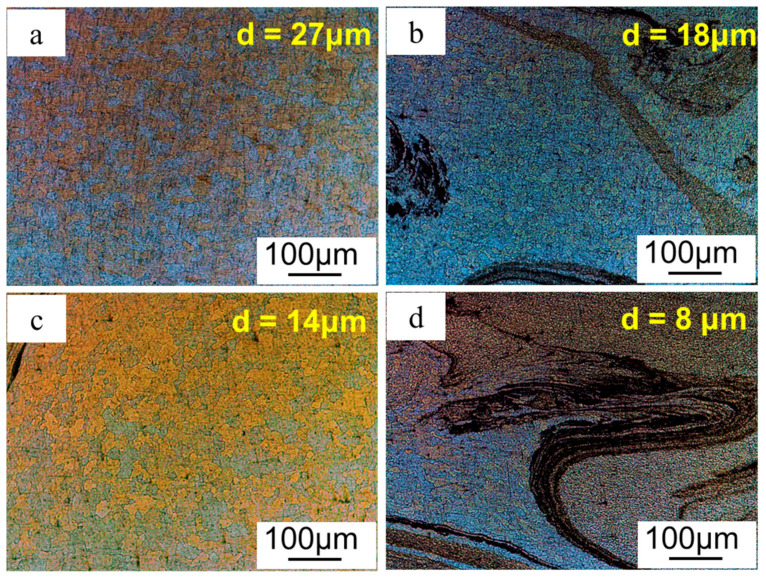
Microstructure of GNPs/Al under different ratios; (**a**) 0 vol.%, (**b**) 0.5 vol.%, (**c**) 1.0 vol.%, and (**d**) 2.0 vol.%.

The microstructure of GNPs/Al under different graphene contents is shown in Figure 8, where the stirring pass was four, and the ratio of stirring/welding velocity was 1000/40. Corresponding grain sizes with 0, 0.5 vol.%, 1.0 vol.%, and 2.0 vol.% were 27 μm, 18 μm, 14 μm, and 8 μm. It was shown that the grain size decreased with the increase in graphene content. Moreover, grain size at the graphene enrichment zone was smaller than that at the graphene-poor zone. Graphene particles can act as heterogeneous cores during the recrystallization process, promoting non-uniform nucleation and thus refining the grain size. Meanwhile, graphene particles are pinned at aluminum grain boundaries, hindering grain boundary movement and thus inhibiting grain growth. According to the Hall–Petch relation, fine grain can improve material strength.

#### 3.2.2. Interface Reaction Evolution

**Figure 9:**
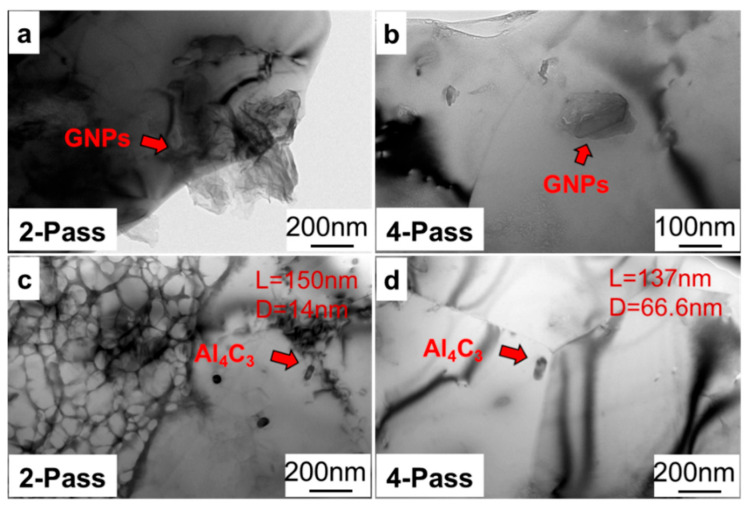
The TEM morphology of GNPs/Al under different passes: (**a**,**c**) two passes and (**b**,**d**) four passes.

Firstly, the interface characteristics of GNPs/Al under different passes were revealed with TEM. As shown in Figure 9a,b, with the increase in stirring pass, the dispersion of graphene in the matrix became more uniform, and the agglomeration phenomenon decreased. As shown in Figure 9c,d, it was found that there was an Al_4_C_3_ phase in the stirring zones, which showed that an interface reaction occurred. Moreover, when the stirring passes increased from 2 to 4, the diameter of the Al_4_C_3_ phase increased from 14 nm to 66.6 nm. This is mainly because more heating input under four passes promoted an interface reaction.

**Figure 10:**
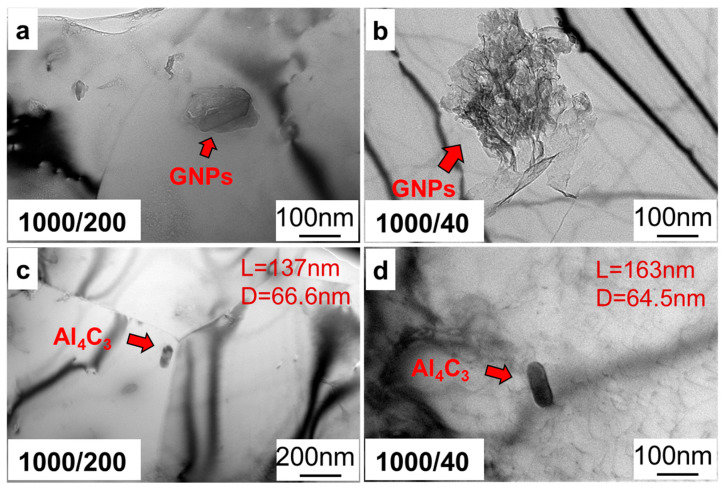
The TEM morphology of GNPs/Al under different ratios of stirring/welding velocity; (**a**,**c**) 1000/200 and (**b**,**d**) 1000/40.

Then, the effect of the ratio of stirring/welding velocity on the interface reaction was characterized by TEM, as shown in Figure 10. As shown in Figure 10a,b, as the ratio of stirring/welding velocity increased, the thickness of the graphene layer decreased, indicating that the dispersion of graphene improved with the increase in the ratio of stirring/welding velocity. As shown in Figure 10c,d, there was also an Al_4_C_3_ phase in the matrix, which showed that the graphene and aluminum matrix underwent an interfacial reaction. Meanwhile, the length of Al_4_C_3_ phase varied from 137 nm to 163 nm. This proves that the increase in the ratio of stirring/welding velocity could intensify the interface reaction between graphene and the aluminum matrix, which was mainly due to the fact that the heat input under the ratio of 1000/40 during processing was higher than that under the ratio of 1000/200.

**Figure 11:**
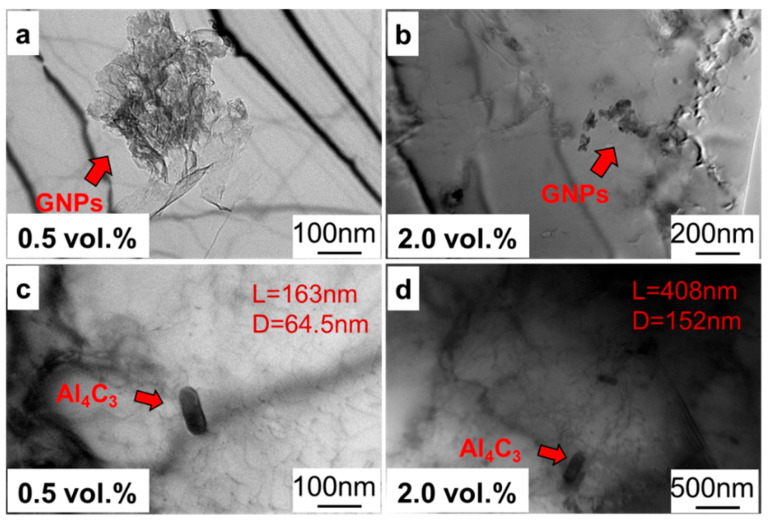
The TEM morphology of GNPs/Al with graphene content; (**a**,**c**) 0.5 vol.% and (**b**,**d**) 2.0 vol.%.

Finally, the interface morphology under different graphene contents was observed, as shown in Figure 11. As shown in Figure 11a,b, with the increase in graphene content, the thickness of graphene layer increased, and the dispersion of graphene decreased. As shown in Figure 11c,d, it was found that with the increase in graphene content, the diameter of the Al_4_C_3_ phase varied from 64.5 nm to 152 nm, and the length varied from 163 nm to 408 nm. It could be concluded that the interface reaction between graphene and the aluminum matrix could be intensified by improving the volume fraction of graphene. The main reason is that the nucleation sites of the Al_4_C_3_ phase were generally at the defects of the graphene structure, the beginning of the carbon nanotubes, etc. As the graphene content increased, the nucleation sites of the Al_4_C_3_ phase increased, promoting the nucleation and growth of the Al_4_C_3_ phase.

### 3.3. Discussion 

The main strengthening mechanism of aluminum alloys includes fine-grain strengthening, solid solution strengthening, precipitation strengthening, and work hardening. After introducing the graphene particle reinforcement phase, there is a load transfer of graphene, which leads to load transfer strengthening. Meanwhile, there is a mismatch of the thermal expansion coefficient between graphene and the aluminum matrix, which also makes a contribution to strengthening and is called thermal expansion mismatch strengthening.

GNPs/Al under four passes + stirring/welding velocity ratios of 1000/40 + 2.0 vol.% conditions were selected in order to discuss the strengthening mechanism. As shown in Figure 5, the ultimate strength of GNPs/Al was 119.2 MPa, while the ultimate strength of aluminum without graphene was 70 MPa, which shows that the ultimate strength increased by 49.2 MPa under the action of graphene.

For fine-grain strengthening, as shown in Figure 8, the grain size of GNPs/Al was 8 μm, while the grain size of the specimen without graphene was 27 μm. The strengthening contribution due to the fine grain caused by graphene is expressed as follows:

(1) $$\Delta \sigma_{\mathrm{grain}}=k_{y}(\frac{1}{\sqrt{d}}\;-\frac{1}{\sqrt{d_{0}}})$$

where d is the grain size of GNPs/Al (8 μm), $d_{0}$ is the grain size of the original material (27 μm), and $k_{y}$ is the ratio coefficient. For the aluminum alloy, the $k_{y}$ is 0.04 MPa $\sqrt{m}$, and the calculated $\Delta \sigma_{\mathrm{grain}}$ is 6.4 MPa, which makes a 13% strength contribution to the strength increment caused by the graphene.

For load transfer strengthening, hard particles can bear more external loads than the soft matrix during tension, which is described with a shear lag model [19] and Eshelby model [20], expressed as follows:

(2) $$\Delta \sigma_{\mathrm{LT}}=\frac{f\sigma_{m}}{2}$$

where $\sigma_{m}$ is the matrix strength ($\sigma_{m}=$ 52 MPa), and f is the volume fraction (f = 2%). The calculated $\Delta \sigma_{\mathrm{LT}}$ is 0.52 MPa, which makes a 1% strength contribution to the strength increment caused by the graphene.

For precipitation strengthening or Orowan strengthening [21], graphene particles are regarded as a precipitate to prevent dislocation movement. This can be modeled by the following:

(3) $$\Delta \sigma_{\mathrm{Orowan}}=\frac{0.81MGb}{2\pi \sqrt{1-v\left(\lambda -d_{t}\right)}}\ln\left(\frac{d_{t}}{b}\right)$$

where M is the Taylor factor (M=3.06), G is the shear modulus (G=26.2 GPa), v is the Poisson ratio (v = 0.33), b is the Burgers vector (b = 0.286 nm), $d_{t}$ is the average size of graphene ($d_{t}=2$00 nm), and λ is the graphene interlayer particle spacing. According to reference [22,23,24], the λ is expressed as follows:

(4) $$\lambda =\sqrt{\frac{2}{3}}\left(\sqrt{\frac{\pi }{f}\;}-2\right)\frac{d_{t}}{2}$$

According to Equations (3) and (4), the calculated $\Delta \sigma_{\mathrm{Orowan}}$ is 35.8 MPa, which makes a 72.7% strength contribution to the strength increment caused by the graphene.

For thermal expansion mismatch strengthening, the thermal expansion coefficients of the Al matrix and graphene are 2.3 × 10^−5^ °C and 6 × 10^−6^ °C, respectively. The mismatch of thermal expansion coefficients produces high-density dislocation caused by plastic deformation during the FSP, which can cause a strength contribution to the Al matrix, as expressed in [25,26,27].

(5) $$\sigma_{\mathrm{CTE}}=\alpha Gb\sqrt{\frac{6\Delta T\Delta Cf}{bd_{t}\left(1-f\right)}}$$

where α is the ratio constant (α = 1.25 for aluminum alloy), ΔT is the difference of preparation temperature and room temperature, and ΔC is the thermal expansion coefficient difference of the Al matrix and graphene. The calculated $\sigma_{\mathrm{CTE}}$ is 27.68 MPa, which makes a 56.2% strength contribution to the strength increment caused by the graphene.

According to the above strength theory and calculated strength values, each strength contribution was added up, and the calculated strength should have been 140.4 MPa, which is much higher than the actual 119.2 MPa. The reason for this is as follows. During analysis of the strengthening theory, it was assumed that the graphene was uniformly sized and dispersed and that the graphene exhibited a two-dimensional planar structure in the matrix without wrinkles. However, as shown in Figure 1 and Figure 9, the graphene had certain wrinkles, and its dispersion needed to be improved, which weakened the actual strengthening effect. In addition, the presence of the Al_4_C_3_ phase proved that the graphene and aluminum matrix underwent an interfacial reaction, as the Al_4_C_3_ phase is a brittle phase that can reduce the mechanical properties of the composites. Therefore, the interface reaction and the graphene dispersion significantly influenced the mechanical properties of GNPs/Al. Meanwhile, it can be inferred that controlling graphene morphology and suppressing interface interaction are effective methods to improve the performance of GNPs/Al, which can be studied further in future studies.

In summary, during the preparation of GNPs/Al with FSP, plastic strain, heat input, and graphene content were key influencing factors. Large degrees of plastic strain can make graphene with good dispersion by increasing the stirring passes, which can improve the strength of GNPs/Al. Low heat input limits the plastic flow of graphene in a matrix, while excessive heat input can enhance the interfacial reaction and produce more brittle phase, Al_4_C_3_, which is mainly related to the stirring velocity and welding velocity. Graphene content is the strengthening base of GNPs/Al, which not only refines the grain size but also improves the load transfer ability, and it improves the material’s performance. In future work, the question of how to suppress interface reactions and improve the dispersion of graphene needs further study.

## 4. Conclusions

This paper shows that FSP is an effective method to prepare a graphene-reinforced aluminum matrix composite. The corresponding effect of process parameters on GNPs/Al was deeply studied, mainly including stirring pass, the ratio of stirring/welding velocity, and graphene content. The main conclusions are as follows:

(1) As the ratio of stirring/welding velocity increases and the graphene content decreases, the grain size increases, which is due to higher heating input and less heterogeneous cores, respectively. However, the stirring pass has no significant impact on grain size due to the dynamic equilibrium between grain growth and dynamic recrystallization.
(2) By controlling the heat input and plastic strain during FSP, the tensile strength of GNPs/Al can be improved with the increase in the stirring pass and the selection of the appropriate ratio of stirring/welding velocity. Under appropriate process parameters, the interface reaction and dispersion of graphene need to be adjusted and balanced.
(3) A high graphene content can improve the material’s strength by refining the grain size, improving the load transfer ability, and acting as a precipitate to prevent dislocation movement. It should be noted that high graphene content can decrease elongation due to the poor dispersion of graphene and the interface reaction between graphene and an aluminum matrix.

## Figures and Tables

**Table 1:** The chemical composition of the 1060 aluminum alloy sheet.

Composition	Si	Fe	Cu	Mn	Mg	Zn	Ti	Al
wt.%	0.25	0.035	0.05	0.03	0.03	0.05	0.03	Bal.

**Table 2:** The main mechanical properties of the 1060 aluminum alloy sheet.

Elastic Modulus	Yield Strength	Ultimate Strength	Elongation	Hardness
70 GPa	64 MPa	82 MPa	21%	26 HV

**Table 3:** The designed preparation process matrix of the GNPs/Al.

Pass Number	Stirring Velocity (rpm)	Welding Velocity (mm/min)	Graphene Content (vol.%)
1	1000	200	0.5
2			
3			
4			
4	400	40	0.5
4	1000	40	0.5
			1
			2
			0

## Data Availability

The original contributions presented in the study are included in the article, further inquiries can be directed to the corresponding author/s.
